# Salivary IL-6 Concentration Is Associated with Frailty Syndrome in Older Individuals

**DOI:** 10.3390/diagnostics12010117

**Published:** 2022-01-05

**Authors:** Pablo Gómez-Rubio, Isabel Trapero, Omar Cauli, Cristina Buigues

**Affiliations:** 1 Department of Nursing, University of Valencia, 46010 Valencia, Spain; pagorubio1@hotmail.com (P.G.-R.); isabel.trapero@uv.es (I.T.); cristina.buigues@uv.es (C.B.); 2 Frailty and Cognitive Impairment Research Group (FROG), University of Valencia, 46010 Valencia, Spain

**Keywords:** salivary biomarkers, frailty, IL-6, fatigue, weight loss, physical activity

## Abstract

Background: One of the physiological changes that is most closely associated with frailty is the increase in pro-inflammatory cytokines, and IL-6 in particular. Most studies have demonstrated this association using blood samples. We analyzed the relationship between frailty syndrome, individual frailty criteria, and IL-6 levels obtained by saliva tests. Methods: A cross-sectional pilot study was performed among women institutionalized in nursing homes. Frailty was defined as having three or more of the following components: low lean mass, weakness, self-reported exhaustion, low activity level, and slow walking speed; prefrailty was defined as having one or two of those components. Results: There was a significant and positive correlation between the frailty score and salivary IL-6 concentration. Regarding the associations between IL-6 and individual dichotomized frailty criteria, there were significant differences in salivary IL-6 concentration in two frailty criteria: weight loss (*p* = 0.002) and low physical activity (*p* = 0.007). Receiver operating characteristic curve analysis showed that IL-6 concentration significantly (*p* < 0.05) (although moderately) discriminated patients that progressed in the frailty syndrome (the area under the curve value was 0.697 with 95% CI 0.566–0.827). Conclusions: Salivary IL-6 concentration can be used as potential biomarker of frailty syndrome and as a tool to monitor the effects of interventions in frail individuals.

## 1. Introduction

Frailty is a crucial age-related clinical syndrome that is commonly defined as a state of increased vulnerability to external stressors, and occurs as a consequence of the cumulative decline in many physiological systems over a lifetime [1]. This syndrome describes the physical and functional decline that occurs as a consequence of certain diseases (e.g., cancer and chronic infection) but which can also occur in the absence of disease [2]. In recent years, frailty has been recognized as an emerging public health priority and the need for early recognition and intervention for frailty is pressing [3]. This is because it is widely accepted as a critical issue for aging populations worldwide, as it is relevant to multiple adverse outcomes, including hospitalization, institutionalization, and premature mortality [4].

An internationally accepted definition of frailty has still not been reached despite the importance of this clinical syndrome. The most commonly used definition considers frailty to be a phenotype comprising five indicators: unintentional weight loss of 5% or 10 pounds within one year, slow gait speed, reduced grip strength, exhaustion, and physical inactivity. Frailty is clinically diagnosed by the presence of three or more of these indicators and is preceded by a prodromal stage known as prefrailty, defined by having one or two indicators [5].

The pathophysiological changes underlying and preceding frailty are not clearly known. The etiology of frailty is not well understood, but it has been associated with changes in several physiological systems, including inflammation, coagulation, hematological, and endocrine systems [6]. Inflammation is one such potential pathophysiological change, which may be closely linked with frailty [7]. Pro-inflammatory cytokines may influence frailty either directly, by promoting protein degradation, or indirectly by affecting important metabolic pathways [8]. Researchers have reported that increased levels of inflammatory markers, such as interleukins-6 (IL-6), tumor necrosis factor-α (TNF -α), and C-reactive protein (CRP) are associated with frailty syndrome [9]. In particular, several studies have found a significant association between IL-6 in blood and frailty syndrome in older individuals [10,11,12,13,14,15,16]. The fact that IL-6 stimulates CRP synthesis and inhibits TNF-α, may be one of the reasons why it has received special attention in the scientific literature. IL-6 is synthesized from mononuclear phagocytes, vascular endothelial cells, fibroblasts, and adipose and muscle tissues. Its concentration is modulated by numerous factors, including proinflammatory agents, viral and bacterial pathogens, neurotransmitters and second messengers, and its function is stimulation of neutrophil production by bone marrow progenitors, promoting synthesis of proinflammatory cytokines and inhibiting TNF-α [17,18]. Several studies have reported evidence for the role of IL-6 in the inflammatory processes through which the immune system intercommunicates with the central nervous system (CNS). It has been observed that IL-6 levels increase with age and that high levels are associated with the cognitive decline that occurs in old age [19,20,21]. In addition, IL-6 levels inversely correlate with hippocampal grey matter volume [22] as well as with the integrity of the brain white matter [23]. Interactions between IL-6 and the CNS are one of the mechanisms by which the immune system influences the performance of cognitive functions, and these also play a role in neurodegeneration and cognitive functionality [24].

Owing to its easy application in clinical practice and prognostic value in therapeutic decisions in medical specialists, the identification of a frail status is essential in older adults. Biomarkers may help identify patients who are at a risk of becoming or being frail [10]. Biomarkers provide objective, measurable indices for normal and/or pathogenic states as well as responses to therapeutic interventions [25]. They are useful for detecting subclinical changes that lead to observable indicators of frailty and testing intervention protocols for frailty, as they reflect changes in physiological processes that underlie and/or relate to frailty [26].

In order to better understand the etiology, time course, and consequences of inflammatory processes and to develop better interventions, there is a need for new methods for assessing inflammation that can be conducted in ecological contexts, across time, and at multiple moments throughout the day [27]. Blood has been the usual biological fluid in which many analytes are measured for clinical and research purposes. This is reasonable, because blood is a systemic fluid that reaches all parts of the body and can influence the biological behavior of tissues or on the other hand, receive and transport tissue-derived components that reflect what is taking place in bodily tissues [28]. Although the detection of serum and blood biomarkers is a standard practice, the use of noninvasive samples is desirable.

When studying other methods, some authors have reported that saliva is a potential diagnostic tool because of its ease of collection and noninvasiveness. [29]. Human saliva contains proteins, peptides, hormones, and enzymes, each of which can easily be used to assess different diseases [30]. Approximately 1400–2000 proteins have been identified in saliva [31]. The number of proteins in saliva shows its diversity, and each of these proteins can be used as a simple tool to assess toxicity, infection, and immunological and hormonal levels. Human saliva represents whole body images and is also known as the “mirror of the body”. A biomarker is a measurable nano-protein, which is used to predict a biological state [32]. Some studies have analyzed salivary IL-6 levels in patients not only with common oral pathologies [33], but also in systemic diseases such as polycystic ovary syndrome [34], bladder pain syndrome sleep disorders [35], in sepsis [29], Huntington’s disease [36], diabetes [37], in stroke [38], Hashimoto’s disease [39], and inflammatory diseases [40]. However, the association between salivary IL-6 levels in patients with frail syndrome is unknown. There is a consensus about the role of blood IL-6 levels and its correlation with frailty syndrome in older individuals. This study aims to analyze the relationship between frailty syndrome and IL-6 levels obtained through saliva tests.

## 2. Materials and Methods

### 2.1. Study Population

A cross-sectional study with a residential profile was conducted in 2019, among institutionalized older individuals living in long-stay centers (GeroResidencias La Saleta, Valencia, Spain) in the province of Valencia (Spain). Exclusion criteria were dementia, severe psychiatric illness (schizophrenia, bipolar disorder, etc., which will have not permitted answering to questions) or blindness (which will have not permitted the measurements of all frailty criteria). In order to rule out the contribution of known inflammatory processes to modify IL-6 levels, residents with acute infections requiring antibiotic treatment, known cancer, inflammatory and autoimmune diseases were also excluded. None of the patients were receiving corticoid or immunosuppressive drugs at the time of saliva sampling.

This research was conducted according to the requirements of the Declaration of Helsinki, and the entire study protocol was approved by the local ethical committee. Written informed consent was obtained from all participants. We measured the socio-demographic characteristics, and the five frailty criteria (involuntary weight loss, low energy or exhaustion, slow mobility, muscle weakness, and low physical activity) based on Fried’s frailty criteria [5], and performed geriatric assessments using the Tinetti index, mini-mental score examination (MMSE) test, Yesavage scale, Barthel index, and Athens insomnia scale (AIS) to estimate the severity of insomnia.

### 2.2. Measurement of Frailty Criteria

Frailty was measured by assessing the presence or absence of five measurable criteria of the frailty syndrome (Fried criteria [5]) which were defined as follows:

(1) Weight loss. Weight loss was defined as the unintentional loss of 4.5 kg or more in the past year.
(2) Self-reported exhaustion. Exhaustion was indicated if the subjects responded positively to either of the statements on the Centre for Epidemiological Studies depression scale [41]: “I felt that everything I did took a lot of effort at least three or four days a week” and “I felt that I could not keep doing things at least three or four days a week”, when referring to the previous week, or if the subject answered “Often” or “Most of the time” to the question “How often in the last week did you feel that everything you did was an effort?”, which is also included on the Centre for Epidemiological Studies depression scale [42]. All burnout criteria were estimated to be present.
(3) Physical activity (PA). Low PA was quantified using the Spanish adaptation of the Minnesota Leisure Time Physical Activity Questionnaire (MLTPAQ) readapted for women [43,44]. The MLTPAQ was administered by a trained interviewer who was provided with precise instructions and a detailed list of PA. The participants received a list of activities and were asked to tick those they had performed during the past year. To avoid recall bias as much as possible, activities performed in the last week were compiled first, followed by those performed in the last month, the last quarter and finally the last year, always including the first periods. For validation purposes, only the information referring to the last year was used. The total leisure time PA energy expenditure (EEPA) was calculated from this questionnaire and used to quantify PA [45].
(4) Slowed motor performance. Slowness was assessed using the 4–6 m walking speed test, adjusted for sex and height according to the standards of the Brief Physical Performance Battery [46]. A low walking speed was estimated to correspond to the worst quintile for sex and height of the group.
(5) Weakness. To determine weakness, strength was measured with a Jaymar hydraulic dynamometer, according to the standards of the Hispanic Established Populations for the Epidemiologic Studies of the Elderly [47].

People who met three or more criteria were classified as frail, those who met 1 or 2 were pre-frail, or those who did not meet any of the criteria were robust.

### 2.3. Measurement of IL-6

Saliva samples were collected from the patients described above in the morning, with at least 2 h of fasting (between 9–11 h in the morning) using the Salivette^®^ Cortisol system (Sarstedt, Germany). 100 μL of each sample were centrifuged and the supernatant was aliquoted into Eppendorf tubes and frozen (−80 °C) until further analysis. The samples were brought to room temperature, and immunoassay ELISA analysis was performed using the High Sensitivity Human Elisa Kit for IL-6 (AbCam ab46042) according to the manufacturer’s instructions. Changes in color intensity and the absorbance at 450 nm and 490 nm was read using the ELISA microplate reader, and a standard curve was prepared by plotting absorbance readings of standards against their concentrations using the Graphpad program.

### 2.4. Statistical Analysis

The continuous variables were expressed as the mean and standard deviation, and the categorical variables as the absolute value with their percentage. The normal distribution of each variable was assessed with the Shapiro–Wilk test in order to determine whether a parametric or non-parametric test should be applied. The correlation between quantitative variables was determined by Spearman’s (for non-normal data distribution) or Pearson’s (for normal data distribution) correlation test. The differences between the two groups were analyzed with the non-parametric Mann–Whitney U test or the parametric Student t-test. We evaluated linear correlations between continuous variables using the Spearman’s rank test. The discrimination accuracy of the predictive model was calculated using C-statistics (area under the receiver operating characteristic curve; AUC). Statistical significance was set at a *p*-value of less than 0.05. All statistical analyses were performed using the SPSS software package (version 24.0; SPSS, Inc., Chicago, IL, USA).

## 3. Results

### 3.1. Study Population, Frailty Score, Geriatric Assessment

A total of 72 subjects (84.7% female) living in nursing care centers located in the province of Valencia (Spain) were recruited for the study. A total of 8.5% of the patients were found to be robust, 54.9% were prefrail, and 36.6% were frail. The most common Fried’s criterion in the sample was poor physical activity (75%) followed by slow walking speed (68.1%), exhaustion (29.2%), weakness (22.5%), and weight loss (8.3%). The psycho-geriatric assessments are shown in Table 1.

### 3.2. Evaluation of the Relationship between Salivary IL-6 Concentration and Geriatric Scales and Frailty Criteria

IL-6 was not significantly associated with any of the geriatric assessment scales (Tinetti gait index: *r* = 0.18; *p* = 0.12; Tinetti balance index: *r* = 0.22; *p* = 0.064, Spearman); Barthel index to measure the activities of daily living and mobility (*r* = 0.91; *p* = 0.44, Spearman); MMSE test for cognitive impairment (*r* = −0.15; *p* = 0.20); Yesavage scale for geriatric depression (*r* = −0.23; *p* = 0.08, Spearman); or the Athens insomnia scale for severity of insomnia (*r* = 0.17; *p* = 0.14, Spearman).

No significant correlations were observed between IL-6 count and self-reported exhaustion (*r* = 0.23; *p* = 0.848, Spearman), hand grip strength (*r* = 0.13; *p* = 0.280, Spearman), or slowed motor performance (*r* = 0.13; *p* = 0.267, Spearman).

**Figure 1:**
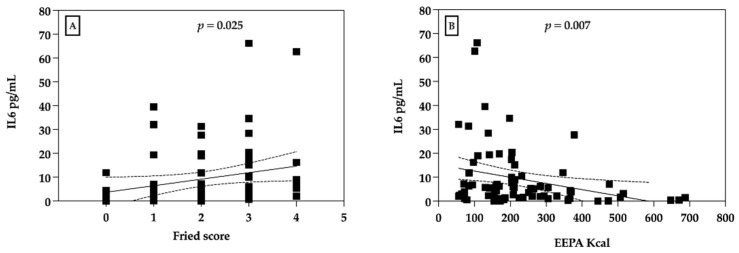
Correlation between IL-6 and Fried score and physical activity. (**A**) Correlation between IL-6 concentration in saliva and frailty score. (**B**) Correlation between IL-6 concentration in saliva and total leisure time PA energy expenditure (EEPA).

However, there was a significant and positive relationship between the number of fulfilled frailty criteria with IL-6 (*r* = 0.26; *p* = 0.025, Spearman) (Figure 1A) and a negative relationship between the specific physical activity criteria (*r* = −0.31; *p* = 0.007, Spearman) (Figure 1B).

There were no significant differences (*p* = 0.07, Kruskal–Wallis) for the IL-6 count between different levels of frailty. There were no significant differences between IL-6 values and sex (*p* = 0.496, Mann–Whitney U) and no significant correlation between IL-6 values and age (*r* = −0.053; *p* = 0.658).

**Figure 2:**
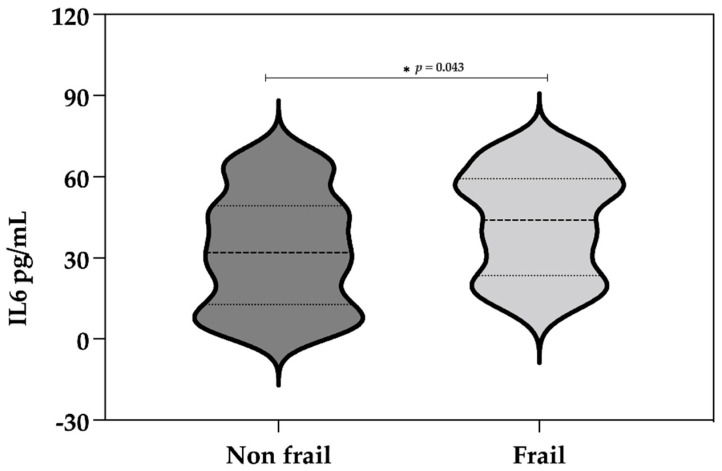
Difference between frailty syndrome and IL-6 (pg/mL). Robust and pre-frail patients were grouped into the non-frail group. Frail patients were those who met 3 or more of the frailty criteria.

There were significant differences between robust and frail individuals in the concentration of salivary IL-6 (*p* < 0.043, Mann–Whitney U) (Figure 2).

**Figure 3:**
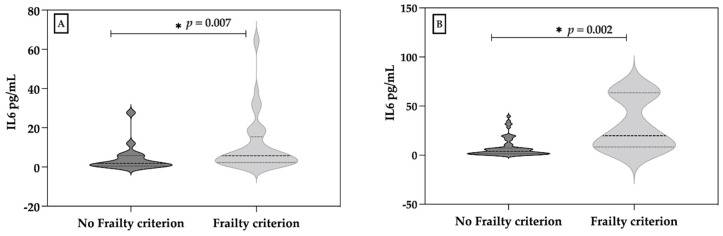
(**A**) Difference between physical activity frailty criteria and (**B**) IL-6 (pg/mL) and weight loss frailty criteria and IL-6 (pg/mL).

For each of the frailty criteria, there were significant differences in IL-6 between those subjects who met the frailty criterion related to the reduction in physical activity and those who did not (*p* = 0.007, Mann–Whitney U) (Figure 3A). Regarding the frailty criterion “Involuntary weight loss” there was a significant difference in salivary IL-6 concentration between the individuals that fulfilled this criterion compared to those did not (*p* = 0.002, Mann–Whitney U) (Figure 3B).

**Figure 4:**
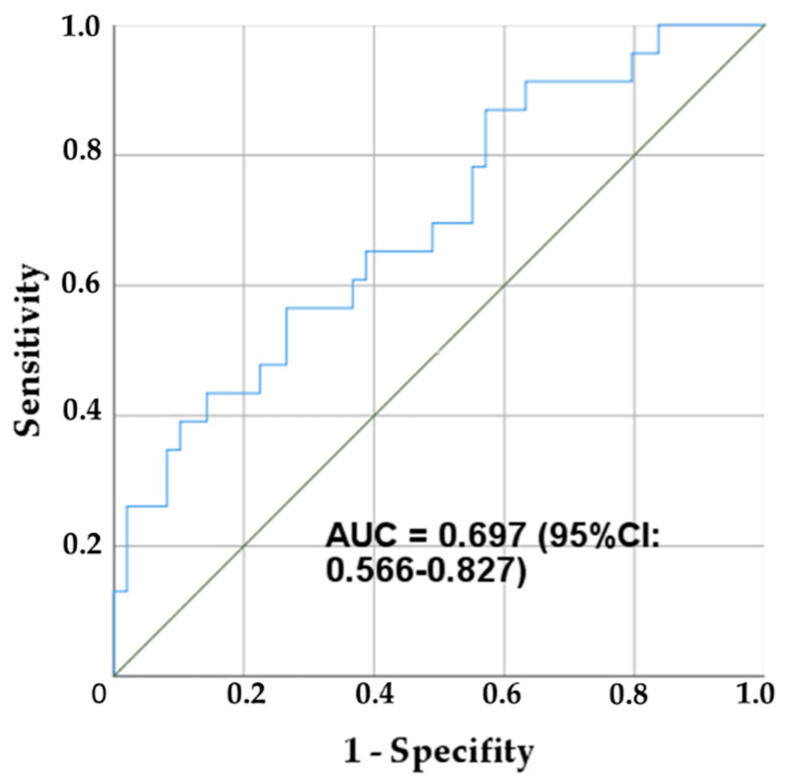
Receiver operating characteristic (ROC) curve for IL-6 concentration and the ability to discriminate between frail individuals versus robust plus pre-frail individuals.

In order to analyze the sensitivity and the specificity of salivary IL-6 concentration to discriminate frail versus non frail (robust) individuals, we performed the receiving operating curve (ROC) analysis (Figure 4). The area under the curve value was 0.697 with 95% CI (0.566–0.827) and the cut-off value 3.74 pg/mL has a sensitivity of 69.0% and a specificity of 52.8%.

## 4. Discussion

Frailty has been recognized an emerging public health priority [3]. In the development of the investigation of frailty, many authors have concluded that inflammation is a potential pathophysiological change, which may be closely linked with this syndrome [7]. Fried hypothesized that high levels of cytokines may induce skeletal muscle loss and aggravate neuroendocrine dysregulation, resulting in the muscle loss and weakness seen in the frailty phenotype [5]. This study showed that the IL-6 levels in saliva were higher in participants classified as frail. This is consistent with the observation by other authors who reported a higher IL-6 serum concentration associated with frailty [48]. Researchers have indicated that increased levels of inflammatory markers such as IL-6, TNF-α, and CRPin blood are associated with reduced muscle strength, poor physical function, frailty, and mortality [49,50,51]. Several studies have reported evidence for the role of IL-6 in the inflammatory processes through which the immune system intercommunicates with the central nervous system (CNS) [52]. Interactions between IL-6 and the CNS are one of the mechanisms by which the immune system influences the performance of cognitive functions, and these also play a role in neurodegeneration and cognitive functionality. It is well established that IL-6 is a potential biomarker for frailty, which could be evaluated as a target for intervention [53] and is considered a predictive marker for incidents of frailty [54].

Regarding to each of the frailty criteria, in this study we demonstrated for the first time that there are significant differences in salivary IL-6 between subjects who are frail compared to pre-frail and robust ones. In addition, and in particular, the frailty criterion regarding reduction in PA was significantly associated with changes in salivary IL-6 concentration in individuals that met this criterion compared with those that did not meet the criterion. There is a significant body of evidence in the literature regarding levels of inflammatory cytokines in IL-6 people and the level of regular exercise in their daily life. In fact, at all ages, individuals engaging in regular physical activities show lower systemic inflammatory markers in blood, including lower baseline IL-6 levels [55]. A negative association between the amount of physical activity scores and the levels of plasma IL-6 has frequently been reported, so that the more PA they perform the lower their levels of IL-6, and conversely, high levels of plasma IL-6 are related to physical inactivity [56]. The activity generated in the skeletal muscles by exercise is known to be one of the main mechanisms by which the plasma and baseline levels of IL-6 can be modulated [57,58]. IL-6 is also known to induce hepatic glucose output [59] and to induce lipolysis [60]. Physical exercise in itself is a stressor which produces inflammation and induces different immune responses associated with the production of interleukins, thereby inducing anti-inflammatory responses. Maintaining a good balance between pro- and anti-inflammatory cytokines may be an important mechanism through which exercise exerts its immunoprotective and immunoregulatory effects. Furthermore, new genetic studies suggest that these effects of exercise may activate both genes involved in the production of leukocytes and those that downregulate inflammation [61]. Several lines of evidence have demonstrated that physical exercise is associated with a systemic reduction in inflammation [62]. A study performed in older men aged 65–74 years demonstrated that regular PA, independent of disease and disability, alter the levels of pro-inflammatory cytokines in blood [63] i.e., the very physically active group has lower levels of IL-6 than the less active group. In a sample of healthy men with a range of physical activity profiles including older people, this variable was shown to be associated not only with anthropometric variables such as body fat percentage, lean mass, and mass, but also with plasma IL-6 concentration. In addition, age showed an indirect effect on IL-6 through body composition, but no direct effect. The results suggest that PA improves the inflammatory profile through the improvement of body composition, but that there are other pathways as well [64].

Another criterion of frailty in which significant differences were found in this study was weight loss. Interleukin-6 (IL-6) is a cytokine implicated in the regulation of energy metabolism [65]. Elevated circulating levels of IL-6 influence both glucose homeostasis [66] and lipid metabolism [67]. While the effects of IL-6 on glucose homeostasis are conflicting [68], there is greater consensus regarding its role in regulating lipid metabolism. Infusion of IL-6 into healthy individuals stimulates lipolysis and fatty acid oxidation [60]. In vitro studies of adipocytes and myotubes have confirmed the effects of IL-6 on lipolysis and fatty acid oxidation, and evidence shows that IL-6 induces these effects by increasing AMP-activated protein kinase (AMPK) [69]. Finally, studies in rodents support a regulatory effect of IL-6 on adipose tissue mass. Whole-body IL-6 knockout mice develop mature onset obesity and an overall increase in fat mass, a phenotype partially reversed by injection of IL-6 [65].

In this study, for IL-6, the area under the curve value was 0.697 with 95% CI (0.566–0.827) (Figure 3), and the cut-off value 3.74 pg/mL has a sensitivity of 69.0% and a specificity of 52.8%. We demonstrated that higher serum IL-6 levels were significantly associated with the higher levels of frail syndrome. Similar results were found for IL-6 serum concentration [9]. As for IL-6, at a concentration > 1.79 pg/mL, there was a sensitivity of 0.68 and specificity of 0.68 for identification of frail patients, but the sensitivity and specificity were not high.)

Although the results obtained in this study that relate IL-6 with frailty and specifically to levels of PA are well established in the literature, the fact that these results are obtained through saliva suggest this technique can be used as a potential diagnostic tool. Similar results have been obtained in salivary IL-6 values in patients diagnosed with inflammatory diseases such as diabetes [37], sepsis [29] and Huntington’s disease [36]. More recently, an increasing number of studies have suggested that salivary biomarkers can be useful for the diagnosis and follow-up of some diseases or conditions, regardless of their blood concentrations [28]. Some studies have obtained results indicating that salivary IL-6 is highly correlated with blood levels [70]. Saliva testing can therefore be a non-invasive tool for frailty diagnosis, without the sampling technique entailing added stress for the patient which implies changes in levels which could occur as a result of the stress associated with drawing blood [35], and it is inexpensive, can be conducted in ecological contexts across time, and at multiple moments throughout the day [27]. Saliva tests could therefore be used for the diagnosis and to indicate the existence or the risk of developing a disease, as well as the response to a particular therapy. Salivary diagnostics is a rapidly emerging field that is dependent on the development of sensitive and specific biomarkers that can be employed in large scale clinical settings.

The main limitation of our study is the small sample size. Despite it being a homogeneous sample because subjects are institutionalized older individuals living in long-stay centers, and therefore with healthy and controlled habits (no smoking, no alcohol consumption), we have not been able to eliminate the influence of all confounding factors that could modify IL-6 levels. There are mixed reports in the literature about the relationship between salivary IL-6 and dental and periodontal disorders. A lack of correlation between IL-6 concentration in saliva and the presence of periodontal diseases has been already demonstrated. Dogan et al. measured periodontal parameters, including gingival index and plaque index, and did not find any significant association between these parameters and IL-6 concentration in saliva [71]. In contrast, in patients with calcified dental plaque composed of calcium phosphate mineral salts and surrounded by a non-mineralized bacterial layer (called dental calculus), the salivary IL-6 concentration was higher with associated chronic periodontitis [72]. In totally edentulous patients with implant-supported overdentures, the concentration of IL-6 in saliva can be used as markers of peri-implant disease [73]. Significant longitudinal associations between oral health indicators and frailty that highlight the importance of oral health as a predictor of frailty in older age [74]. Frailty syndrome is independently associated with the presence of dental caries and oral status [75]. However, periodontal disease has a weaker association with frailty compared with number of teeth [76], demonstrating no significant association between periodontitis and the frailty criterion grip strength [77,78]. Future research should investigate the role of potential mediating factors in the association between salivary IL-6 and frailty syndrome.” Moreover, our study being a cross-sectional study, does not allow inference about cause–effect relationship between IL-6 and frailty syndrome; therefore, future longitudinal studies with larger samples will be necessary in order to know the relationship and confounding factors.

More research is needed to differentiate the IL-6 levels between frail and pre-frail individuals and acquire an early identification of frail status, essential for clinical practice and the therapeutic decisions to curb the syndrome. It is reasonable to assume that future clinical studies will progressively demonstrate the usefulness of salivary biomarkers related to inflammatory molecules, such as IL-6, for the diagnosis and follow-up of some diseases, and specifically for the diagnosis of the frailty.

In conclusion, this study shows a relationship between frailty syndrome and IL-6 levels obtained through saliva tests. Level of IL-6 is higher in frail individual compared non-frail group as well as it is higher in those subjects who met the frailty criterion related to the reduction in physical activity and the individuals that fulfilled the criterion “Involuntary weight loss” compared to those who did not. These results suggest that salivary IL-6 tests can be useful to distinguish between fragile and non-fragile people and therefore this technique can be used as a potential diagnostic tool.

## Figures and Tables

**Table 1:** Characteristics of the study sample.

Category	Mean Value	Range
Age (years)	83.3 ± 6.9	66–99
Yesavage scale (depressive symptoms)	3.5 ± 3.2	0–11
Athens insomnia scale	4.6 ± 4	0–16
Barthel index (basic activities of daily life)	74.1 ± 22	25–100
Tinetti balance index	10.5 ± 3.6	1–16
Tinetti gait index	8.0 ± 2.8	0–12
Frailty criteria	0 criteria = 6 individuals	
	1 criterion = 18 individuals	
	2 criteria = 22 individuals	
	3 criteria = 19 individuals	
	4 criteria = 7 individuals	

Mean ± standard deviation and range for each value/scale. Geriatric assessment was evaluated by validated scales: Tinetti gait and balance index to determine the risk footfalls, mini-mental score examination test (MMSE), Yessavage scale for geriatric depression, Barthel index to measure the activities of daily living and mobility, Athens insomnia scale (AIS) to estimation of the severity of insomnia.

## Data Availability

Data will be available upon specific request to the corresponding author.
